# Context-dependent interaction between oxytocin gene polymorphisms and alcohol dependence in modulating negative emotions during acute alcohol withdrawal in adult males

**DOI:** 10.3389/fpsyt.2026.1680226

**Published:** 2026-05-15

**Authors:** Zeping Xu, Xiaoying Ye, Guanghui Shen, Yu-Hsin Chen, Xingguang Luo, Huankun Sun, Yimin Kang, Wei Wang, Hua Zhou, Li Chen, Fan Wang, Yanlong Liu

**Affiliations:** 1 Department of Pharmacy, Ningbo Medical Center Li Huili Hospital, The Affiliated Lihuili Hospital of Ningbo University, Ningbo, Zhejiang, China; 2 Cixi Biomedical Research Institute, Wenzhou Medical University, Ningbo, China; 3 Department of Behavioral Medicine, Wenzhou Seventh People’s Hospital, Wenzhou, China; 4 School of Mental Health, Wenzhou Medical University, Wenzhou, China; 5 Department of Psychiatry, Yale University School of Medicine, New Haven, CT, United States; 6 Psychosomatic Medicine Research Division, Inner Mongolia Medical University, Huhhot, China; 7 Beijing Hui-Long-Guan Hospital, Peking University, Beijing, China; 8 Zhejiang Provincial Clinical Research Center for Mental Disorders, The Affiliated Wenzhou Kangning Hospital, Wenzhou Medical University, Wenzhou, China

**Keywords:** alcohol dependence, anxiety, depression, G × E interactions, oxytocin gene polymorphisms

## Abstract

**Objective:**

The importance of multiple gene-environment interaction (G × E) has been highlighted in understanding the etiology of negative emotions. This study examines the impact of oxytocin (*OXT*) polymorphisms (rs2740210, rs6133010, and rs2740209) in combination with alcohol dependence on anxiety and depression symptoms during acute alcohol withdrawal under different social and environmental contexts.

**Method:**

A total of 414 Chinese Han male adults undergoing acute alcohol withdrawal were recruited. Participants provided blood samples for genotyping, self-reported measures of depression and anxiety, assessments of alcohol dependence severity, and demographic information regarding social and environmental contexts.

**Results:**

Results revealed a positive correlation between severity of alcohol dependence and symptoms of depression and anxiety, while oxytocin polymorphism did not have a direct effect on depressive and anxiety symptoms. A significant interaction between *OXT* polymorphism (rs2740210 and rs2740209) and alcohol dependence in relation to anxiety symptoms solely among adults living with family and/or those who were married was observed. Further analyses indicate that the GG and CC genotypes are risk genotypes, while the T allele (rs2740210) and G allele (rs2740209) are non-risk alleles in the interaction between *OXT* genotypes (rs2740210, rs2740209) and alcohol dependence on anxiety among the aforementioned participants.

**Conclusions:**

These findings provide evidence for distinct G × E interaction effects on anxiety and depression symptoms during acute alcohol withdrawal, supporting the weak diathesis-stress model. Furthermore, the study highlights the importance of considering environmental factors when investigating the role of oxytocin as a biological substrate underlying social bonding and the regulation of negative emotions.

## Introduction

Alcohol is perhaps the oldest and most widely used psychoactive substance by humans (1). For instance, in China, the per capita annual consumption of alcoholic beverages in the general population increased from 4.9 liters in 2003–2005 to 7.2 liters in 2016, with regular Chinese drinkers consuming an average of 12.9 liters per capita in 2016 (2). By 2016, China ranked as the second highest drinking country globally, surpassed only by the United Kingdom. Excessive alcohol use, including alcohol dependence and abuse, has significant consequences for social safety, physical health, and mental well-being worldwide, including China (3–5). According to the Global Burden of Diseases, Injuries, and Risk Factors Study, the number of alcohol-related deaths in China increased by 1.82 times between 1990 and 2017, accompanied by an approximately 44% increase in the population attributable fraction (6). Alcohol consumption has been identified as a major contributor to the burden of disease, and excessive alcohol use is associated with mental health issues such as depression and anxiety (3, 7).

There is a growing body of evidence supporting the importance of gene-environment interaction (G × E) in understanding the etiology of excessive alcohol use and negative emotions (8–10). However, the specific details of G × E interactions in relation to the etiology of excessive alcohol use and negative emotions are still largely unknown. Furthermore, existing G × E research has primarily focused on the role of genes in modulating negative emotions resulting from early stressful experiences, such as childhood abuse and maltreatment (11–14). Few studies have examined the interaction between genes and current stressful experiences, such as acute alcohol withdrawal. Moreover, little effort has been devoted to investigating whether these G × E effects are influenced by different social and environmental contexts, which could fully demonstrate the importance of G × E interactions. According to social ecological theory, the interactive effects between biological factors and personal experiences are dependent on the social contexts in which they occur (15).

Negative emotions refer to unpleasant or unhappy feelings experienced by individuals, such as depression, anxiety, loneliness, and anger, which reflect a negative affective response to events or individuals (16). Numerous studies have highlighted the association between alcohol dependence and negative emotions, particularly during the acute withdrawal phase (3, 17, 18). Acute alcohol withdrawal is characterized by a specific set of symptoms that occur when an alcohol-dependent individual abruptly stops or reduces their alcohol consumption. This withdrawal process can trigger a stress response in the brain, leading to heightened levels of anxiety and depression (19, 20). Nonetheless, researchers have observed substantial individual variability in susceptibility to negative emotions during acute withdrawal, suggesting a more complex relationship between alcohol dependence and affective symptoms. Within the framework of G × E interactions, genetic variants are believed to play a critical role in modulating negative emotions during this period.

While prior research has focused primarily on associations between anxiety or depression and polymorphisms in genes such as the serotonin transporter (*5-HTTLPR*) and monoamine oxidase A (*MAOA*) (21), the present study focuses on the potential influence of polymorphisms in the regulatory regions of the oxytocin (*OXT*) gene, including its upstream and downstream flanking regions.

Initially recognized as a hormone involved in lactation (22) and parturition, emerging evidence suggests that OXT also acts as a neuromodulator of negative emotions, including anxiety and depression. The anxiolytic and antistress effects of OXT in rodents were first discovered in the 1990s. For instance, exposure of male rats to novelty, forced swimming, or social defeat leads to a rapid increase in OXT release into the blood as well as in various limbic brain regions, including the paraventricular nucleus and supraoptic nucleus. Additionally, female mice lacking the *OXT* gene exhibit higher levels of anxiety-related behavior compared to their wild-type counterparts in elevated plus maze tests (23). Clinical trials have also indicated a potential relationship between peripheral OXT levels, *OXT* receptor (*OXTR*) gene polymorphisms, and generalized anxiety disorder (24). In a mixed-gender cohort of child and adult patients, plasma and cerebrospinal fluid concentrations of OXT were found to negatively predict trait anxiety scores (25). Furthermore, two single nucleotide polymorphisms (SNPs) of the *OXTR* gene, rs53576 and rs2254298, have been associated with separation anxiety in depressed patients (26). Recent G × E interactions studies have established a robust association between *OXTR* genetic variants and both separation anxiety disorder and general psychopathology. Empirical evidence indicates that exposure to early-life threat moderates the effects of *OXTR* variants, thereby elevating susceptibility to broad-spectrum psychiatric conditions (27–29). These same SNPs have also demonstrated an interaction effect in gene-environment studies on psychopathology.

However, compared to the extensive research on the *OXTR*, relatively few studies have investigated the role of OXT-related variants in psychiatric disorders and the G × E interactions involving OXT. Moreover, given the broad spectrum of negative emotions and the present study’s focus on underlying etiology, this investigation specifically targets clinically significant anxiety and depression symptoms, rather than general or mild mood fluctuations.

Furthermore, it is important to note that most of the existing studies on G × E interactions, including *OXT* polymorphism and the risk for depression or anxiety, have primarily been guided by the diathesis-stress model or the differential susceptibility model. The diathesis-stress model is an early and widely used theoretical model of G × E interaction (30). According to the diathesis-stress model, a stressful environment activates latent diathesis in the form of behavioral, physiological, or genetic predispositions, leading to the expression of psychopathology (31). In addition to innate predispositions, a growing body of work indicates that early exposure to adversity can engender lifelong increases in sensitivity to stressful life events, which heighten the risk for psychopathology (32).

However, an alternative theoretical perspective has proposed the perspective of differential susceptibility to environmental influences as an alternative to the diathesis-stress model. From this perspective, genes that are commonly regarded as susceptible genes are reconceptualized as “plasticity” genes, which are hypothesized to increase responsiveness to different environmental conditions. In other words, individuals with “plasticity alleles” not only suffer from adverse environments but also benefit from supportive environments (33). In the current study, we aim to examine how *OXT* polymorphism and alcohol dependence interact in their associations with depressive or anxiety symptoms to test these two theoretical models. However, due to the lack of relevant empirical findings, we do not have a specific hypothesis regarding this issue.

Even though it is now widely acknowledged that endogenous release or exogenous administration of OXT may facilitate social bonding, emotion regulation, and promote mental health, it has become clear in recent years that these beneficial effects of OXT may occur only under specific circumstances (34). According to the social salience hypothesis of OXT, the effects of OXT are influenced by contextual factors, which are external cues stemming from the environment (e.g., living with a familiar or unfamiliar person) and may influence sensitivity to and interpretation of the emotional significance or salience of a situation (35). The context-dependent effect of OXT is supported by research findings. For example, in a well-validated experiment (35), it was demonstrated that the effects of OXT were determined by social and environmental contexts (i.e., being pro-self or pro-other oriented). OXT administration was shown to decrease cooperation when participants interacted with strangers compared to familiar persons or out-group members compared to in-group members (36). Taken together, the social salience hypothesis of OXT and the aforementioned studies indicate that OXT may increase the salience of safety signals in positive or supportive contexts, which may attenuate stress and decrease negative emotions such as anxiety and depression. Conversely, in unpredictable threatening situations, OXT may trigger orienting responses to threat rather than safe signals and increase anxiety or depression.

This study investigates the interaction effects of three oxytocin gene polymorphisms (rs2740210, rs6133010, and rs2740209) and alcohol dependence on depressive and anxiety symptoms in male adults undergoing acute alcohol withdrawal. The research is guided by a gene–environment interaction framework, focusing specifically on the interplay between *OXT* genetic variation and alcohol dependence severity, and further examines whether these effects are moderated by contextual factors. Based on current theoretical and empirical evidence, the following hypotheses were formulated:

H1: Alcohol dependence severity is positively associated with symptoms of anxiety and depression during acute withdrawal.

H2: *OXT* polymorphism mediates the association between alcohol dependence severity and symptoms of anxiety (H2a) and depression (H2b).

H3: The mediating effect of *OXT* polymorphism on the relationship between alcohol dependence severity and anxiety (H3a) or depression (H3b) is stronger in the presence of positive or familiar social and environmental contexts (e.g., marital status, cohabitation with family) compared to negative or unfamiliar contexts.

Specifically, this study aims to: (1) examine the interactive effects of *OXT* polymorphisms and alcohol dependence on depressive and anxiety symptoms; (2) characterize the nature of this interaction by evaluating both the diathesis-stress and differential susceptibility models; and (3) determine whether the observed G × E effects vary as a function of social and environmental contexts.

## Methods

### Participants

The sample consisted of 414 male adults, aged 20 to 67 years, undergoing acute alcohol withdrawal. Given the low prevalence of alcohol use disorder among females in China, which resulted in an insufficient pool of eligible female participants for recruitment, the present study was conducted exclusively with male participants. Participants were recruited from several hospitals in northern China between 2010 and 2011. All participants were of Chinese Han ethnicity and had been diagnosed with alcohol dependence according to the Diagnostic and Statistical Manual of Mental Disorders, 4th edition (DSM-IV) by trained psychiatrists.

The exclusion criteria included: 1) a history of other drug abuse or dependence (except for nicotine dependence); 2) the presence of serious cardiovascular diseases, liver or kidney disease; 3) a personal or first-degree family history of major psychiatric illness, assessed through a structured clinical interview and a self-reported family history questionnaire at enrollment; 4) lack of a clear understanding of informed consent.

### Sample size determination

An *a priori* power analysis was conducted using the *pwr* package in R (version 4.41). The effect size (*f²* = 0.40) was derived from previous gene–environment interaction research (37). The analysis specified a statistical power of 0.95 (1 − *β*) and a significance level of *α* = 0.05. Results indicated that a minimum sample size of 75 was required for correlation analyses. For regression analyses, assuming an average R² of 0.10 with five predictors, a minimum of 178 participants was needed to achieve adequate power. The final sample of 414 participants therefore exceeded all required thresholds, ensuring sufficient statistical power for the primary analyses.

### Measures

#### Alcohol dependence

Alcohol dependence severity was assessed using the 25-item Michigan Alcoholism Screening Test (MAST) (38). Items are rated on a 4-point scale ranging from 1 (“not at all”) to 4 (“extremely”). The scale demonstrates high internal consistency (Cronbach’s *α* = 0.90). Higher total scores indicate more severe alcohol dependence.

#### Depression

Depressive symptoms were measured using the 20-item Self-Rating Depression Scale (SDS; (39). Each item is rated on a 4-point Likert scale from 1 (“rarely or none of the time”) to 4 (“most or all of the time”). The SDS has strong reliability, with a split-half coefficient of 0.92. Higher scores reflect more severe depressive symptoms.

#### Anxiety

Anxiety severity was assessed using the 20-item Self-Rating Anxiety Scale (SAS) (40). Items are scored on a 4-point Likert scale from 1 (“none or a little of the time”) to 4 (“most or all of the time”). The SAS demonstrates good internal consistency (Cronbach’s *α* = 0.82). Higher scores indicate greater anxiety severity.

#### Social and environmental contexts

Social and environmental contexts were operationalized using marital status and family living circumstances. Participants were categorized as being in a positive or familiar context if they were married or living with family members, and in a negative or unfamiliar context if they were unmarried or living without family.

Marital status and family circumstance were determined using the following two questions: (1) “Are you married?” and (2) “Do you live with your family?” Based on the responses to the first question, participants were divided into two groups: the married group (n = 302, 72.9%) and the unmarried group (n = 112, 27.1%). Based on the responses to the second question, participants were divided into two groups: the living with family group (n = 332, 80.2%) and the living without family group (n = 82, 19.8%).

#### Covariates

The covariates included in the analyses were age and years of education.

### Procedure

This study received ethical approval from the Ethics Committee of Inner Mongolian Medical University. Firstly, a pilot study was conducted with the participation of 30 male adults from the target population to assess the clarity, comprehensiveness, and acceptability of the questionnaires. Secondly, informed consent was obtained from participants prior to data collection. Additionally, participants were asked to complete a series of questionnaires in a quiet ward and provide a blood sample for DNA extraction. All assessments, including self-report questionnaires and blood sampling for genetic analyses, were conducted at least three weeks after admission, following a period of supervised alcohol abstinence. This timing was chosen to ensure that the assessed symptoms of depression and anxiety reflected more stable emotional traits rather than transient states associated with acute alcohol withdrawal syndrome.

### DNA sample collection and genotyping procedures

Genomic DNA was extracted from 5 mL of peripheral blood using the phenol–chloroform method. Genotyping of three single nucleotide polymorphisms (SNPs) in the *OXT* gene (rs2740209, rs2740210, and rs6133010) was performed using TaqMan SNP Genotyping Assays with allele-specific probes from Applied Biosystems. All laboratory procedures were conducted under blinded conditions to minimize bias. Genotype data were successfully obtained for all 414 participants included in the final analysis; reproducibility was confirmed by re-genotyping 10% of samples, which yielded 100% concordance.

### Data analysis

First, the Hardy-Weinberg equilibrium for genotype distributions of each *OXT* genotyping was tested using the χ^2^ test for goodness of fit. Pearson correlation and Spearman’s correlation were conducted to examine the associations among genetic polymorphisms, marital status, family circumstance, age, educational years, alcohol dependence, depression, and anxiety. Then, traditional linear regression was used to provide initial testing for G × E interaction. To assess G × E interactions in different social and environmental contexts, we conducted regression models separated by marital status and family circumstance. When significant interactions were found, we used the region of significance (RoS) analysis to examine the forms of interaction effects. This approach provides potential thresholds for the full distribution of alcohol dependence.

Then re-parameterized regression model was fitted to testing the alternative forms of G × E interaction as follows (41):


$$Y=\{\begin{array}{c} Group:D=0\;B_{0+}B_{1}(X-C)+B_{3}X_{2}+B_{4}X_{3}+E\\ Group:D=1\;B_{0+}B_{2}(X-C)+B_{3}X_{2}+B_{4}X_{3}+E \end{array}$$

Here, *Y* represents the dependent variable of anxiety/depression, and *Group* represents the allelic group. D is a binary variable indicating allelic group membership. For rs2740209, the CC genotype was coded as 0, and CG/GG genotypes were combined into the G-allele group (coded as 1). For rs2740210, the GG genotype was coded as 0, and GT/TT genotypes were combined into the T-allele group (coded as 1). For rs6133010, the AA genotype was coded as 0, and AG/GG genotypes were combined into the G-allele group (coded as 1).

*X* represents alcohol-dependence severity (standardized MAST score). *X_2_* and *X_3_* represent covariates (age and years of education, respectively). *C* denotes the crossover point at which regression lines for the two genotype groups intersect. *B_0_* is the intercept; *B_1_* and *B_2_* are slope coefficients representing the association between alcohol dependence and outcome variables within each genotype group; *B_3_* and *B_4_* are coefficients for the covariates; and *E* is the residual error term (41).

*C* represents the crossover point at which the slopes for the two gene groups intersect. The estimation of the crossover points *C*, along with its confidence interval, distinguishes between the diathesis-stress and differential susceptibility models. If the point estimate and the 95% confidence interval fall at the extreme end of the alcohol dependence scale, the forms of interaction are consistent with the diathesis-stress model. Conversely, if the estimate of *C* falls within the range of alcohol dependence, the forms of interaction are consistent with the differential susceptibility model.

Both the diathesis–stress and differential susceptibility frameworks can be further subdivided into “strong” and “weak” versions. The strong versions posit that only carriers of the “risk/plasticity allele” are sensitive to environmental influences. The weak versions assume that individuals in both genotype groups exhibit environmental sensitivity, but carriers of the “non-risk/non-plasticity allele” show reduced sensitivity compared to carriers of the “risk/plasticity allele”. These models are nested within each other, and an F-test can be used to compare models if one model includes more or fewer parameter estimates than the other. For non-nested models, we compare the Akaike information criterion (AIC) and Bayesian information criterion (BIC) to evaluate which model fits better (41).

#### Transparency and openness

We report how we determined our sample size, all data exclusions, all manipulations, and all measures in the study, and we follow JARS (42). Materials and analysis code for this study are available by emailing the corresponding author. This study’s design and its analysis were not pre-registered.

## Results

### Descriptive statistics

Demographics and clinical characteristics of the study participants, stratified by marital status are presented in **Table 1**. The sample comprised 414 male adults undergoing acute alcohol withdrawal, with a median age of 45 years and a median educational duration of 11 years. The majority of participants were married (72.9%) and resided with family members (80.2%). No significant differences were observed in the genotype distributions of *OXT* polymorphisms between the marital status groups (*p* > 0.05).

**Table 1:** Demographics and clinical characteristics of study participants (N = 414).

Variable	Overall, N = 414^1^	Unmarried, N = 112^1^	Married, N = 302^1^	P-value^2^
Age	45 (37, 50)	43 (29, 48)	45 (39, 50)	0.003
Educational year	11.0 (8.0, 11.0)	11.0 (8.0, 14.0)	11.0 (8.0, 11.0)	0.600
SAS score	34 (28, 40)	34 (27, 40)	34 (28, 40)	0.500
MAST score	9.0 (5.0, 14.0)	11.0 (6.0, 15.0)	8.0 (4.0, 13.0)	0.010
SDS score	61.96 (60.57, 63.06)	61.89 (60.82, 62.92)	61.96 (60.57, 63.06)	0.800
Living style				<0.001
Living without family	82 (19.8%)	54 (48%)	28 (9.3%)	
Living with family	332 (80.2%)	58 (52%)	274 (91%)	
rs2740210				>0.900
G G	200 (48%)	55 (49%)	145 (48%)	
G T	172 (42%)	45 (40%)	127 (42%)	
T T	42 (10%)	12 (11%)	30 (9.9%)	
rs2740209				>0.900
C C	208 (50%)	58 (52%)	150 (50%)	
C G	172 (42%)	45 (40%)	127 (42%)	
G G	34 (8.2%)	9 (8.0%)	25 (8.3%)	
rs6133010				0.700
A A	191 (46%)	55 (49%)	136 (45%)	
A G	44 (11%)	10 (8.9%)	34 (11%)	
G G	179 (43%)	47 (42%)	132 (44%)	

^1^n (%); Median (IQR).

^2^Pearson’s Chi-squared test; Fisher’s exact test; Wilcoxon rank sum test.

Continuous variables are presented as median (interquartile range, IQR) and compared using the Wilcoxon rank-sum test; categorical variables are presented as n (%) and analyzed using Pearson’s chi-square test or Fisher’s exact test, as appropriate.

SAS, Self-Rating Anxiety Scale; MAST, Michigan Alcoholism Screening Test; SDS, Self-Rating Depression Scale.

**Figure 1:**
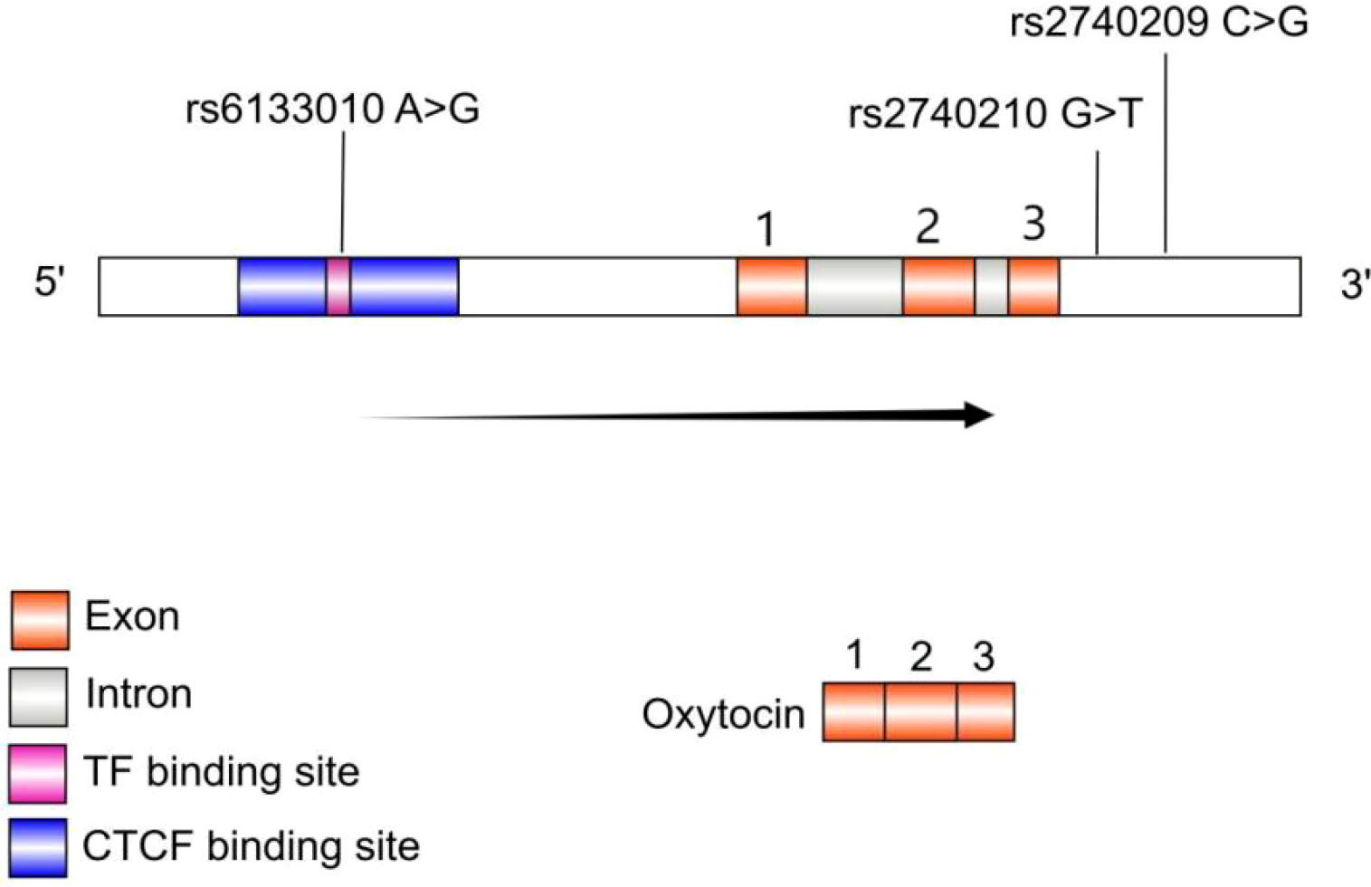
Location and functional prediction of rs6133010, rs2740209, and rs2740210. The genomic structure of the *OXT* gene and the positions of the three SNPs are depicted. The potential functional roles of these SNPs were predicted using the Ensembl Regulatory Build tool. *OXT* rs6133010 is located within a region harboring a putative CTCF-binding site (position 255 in a full-length sequence of 600 nucleotides) and a transcription factor (TF)-binding site (position 9 in a full-length sequence of 25 nucleotides). *OXT* rs2740209 and rs2740210 are situated in the downstream region of the oxytocin transcript.

The genomic structure of the *OXT* gene and the location of the three SNPs are shown in **Figure 1**. The genotype frequencies for *OXT* rs2740210 were GG: 48%, GT: 42%, TT: 10%; for *OXT* rs2740209 were CC: 50%, CG: 42%, GG: 8.20%; and for *OXT* rs6133010 were AA: 46%, AG: 11%, GG: 43%. Hardy-Weinberg equilibrium (HWE) was assessed in the cohort of patients with alcohol dependence. Genotype distributions for *OXT* rs2740210 (*χ*^2^ = 0.31, *p* > 0.05) and *OXT* rs2740209 (*χ*^2^ = 0.03, *p* > 0.05) were consistent with HWE expectations. In contrast, *OXT* rs6133010 exhibited a highly significant deviation from HWE (*χ*^2^ = 256.57, *p* < 0.0001).

**Table 2** presents the correlations of the variables. Neither lifestyle nor marital status was associated with alcohol dependence, anxiety, or depression. Both anxiety (*r* = 0.45, *p* < 0.01) and depression (*r* = 0.22, *p* < 0.01) were positively correlated with alcohol dependence, while education (*r* = -0.18, *p* < 0.01) was negatively correlated with alcohol dependence. *OXT* rs6133010 polymorphism was associated with anxiety (*r* = 0.12, *p* < 0.05); however, the polymorphisms *OXT* rs2740210 and rs2740209 were unrelated to all the other variables. Demographic and clinical characteristics stratified by living situation (living with vs. without family) are presented in **Table 3**.

**Table 2:** Correlation analysis among demographic variables and psychiatric symptoms.

Variables	Lifestyle	Marital status	rs2740210	rs2740209	rs6133010	Age	Educational years	MAST score	SAS score	SDS score
Lifestyle	—									
Marital status	0.38^**^	—								
rs2740210	-0.02	0.04	—							
rs2740209	0.01	0.04	0.94^***^	—						
rs6133010	0.05	0.03	-0.04	-0.07	—					
Age	0.07	0.17^*^	-0.03	-0.03	-0.01	—				
Educational years	0.01	-0.05	-0.02	0.00	0.01	-0.38^**^	—			
MAST score	0.06	-0.1	0.01	-0.01	0.09	0.17^**^	-0.18^**^	—		
SAS score	0.01	0.00	-0.01	-0.04	0.12^*^	0.03	-0.11^*^	0.45^**^	—	
SDS score	0.03	-0.06	0.02	-0.01	0.03	0.00	-0.01	0.22^**^	0.17^**^	—

The values presented are Pearson correlation coefficients. Statistically significant correlations are indicated in bold. Significance levels: *p < 0.05, **p < 0.01, ***p < 0.001.

SAS, Self-Rating Anxiety Scale; MAST, Michigan Alcoholism Screening Test; SDS, Self-Rating Depression Scale.

**Table 3:** Comparisons between living without family and living with family (N = 414).

Variable	Overall, N = 414^1^	Living without family, N = 82^1^	Living with family, N = 332^1^	P-value^2^
Age	45 (37, 50)	45 (32, 49)	45 (39, 50)	0.110
Educational year	11.0 (8.0, 11.0)	11.0 (8.0, 13.0)	11.0 (8.0, 11.0)	0.600
Anxiety	34 (28, 40)	35 (27, 40)	34 (28, 40)	0.700
MAST score	9.0 (5.0, 14.0)	9.0 (4.0, 13.0)	9.0 (5.0, 14.0)	0.700
SDS score	61.96 (60.57, 63.06)	62.23 (60.57, 63.20)	61.82 (60.57, 63.06)	0.700
SAS score				<0.001
Unmarried	112 (27.1%)	54 (66%)	58 (17%)	
Married	302 (72.9%)	28 (34%)	274 (83%)	
rs2740210				>0.900
G G	200 (48%)	40 (49%)	160 (48%)	
G T	172 (42%)	35 (43%)	137 (41%)	
T T	42 (10%)	7 (8.5%)	35 (11%)	
rs2740209				0.600
C C	208 (50%)	39 (48%)	169 (51%)	
C G	172 (42%)	38 (46%)	134 (40%)	
G G	34 (8.2%)	5 (6.1%)	29 (8.7%)	
rs6133010				0.048
A A	191 (46%)	43 (52%)	148 (45%)	
A G	44 (11%)	3 (3.7%)	41 (12%)	
G G	179 (43%)	36 (44%)	143 (43%)	

^1^n (%); Median (IQR).

^2^Pearson’s Chi-squared test; Fisher’s exact test; Wilcoxon rank sum test.

Continuous variables are presented as median (interquartile range, IQR) and compared using the Wilcoxon rank-sum test; categorical variables are presented as n (%) and analyzed using Pearson’s chi-square test or Fisher’s exact test, as appropriate.

SAS, Self-Rating Anxiety Scale; MAST, Michigan Alcoholism Screening Test; SDS, Self-Rating Depression Scale.

### Interactions between *OXT* genotyping and alcohol dependence on anxiety

#### Interactions between *OXT* genotyping and alcohol-dependence on anxiety in the different living styles

We first conducted hierarchical regression analysis to examine the interaction between *OXT* genotyping and alcohol dependence on anxiety in individuals living with family (yes vs. no).

There was a significant main effect of alcohol dependence on anxiety in different living styles. For those living with family, higher alcohol dependence was associated with higher levels of anxiety (|*β*|s = 0.38 to 0.54, *p*(s) < 0.01). Similarly, for those living without family, higher alcohol dependence was also associated with higher levels of anxiety (|*β*|s = 0.42 to 0.50, *p*(s) < 0.01). However, there were no significant main effects of *OXT* genotyping (rs2740210, rs2740209, rs6133010) on anxiety in different living styles. In other words, *OXT* genotyping did not independently contribute to anxiety levels (living with family: |*β*|s = 0.04 to 0.07, *p*(s) > 0.05; living without family: |*β*|s = 0.08 to 0.22, *p*(s) > 0.05).

**Figure 2:**
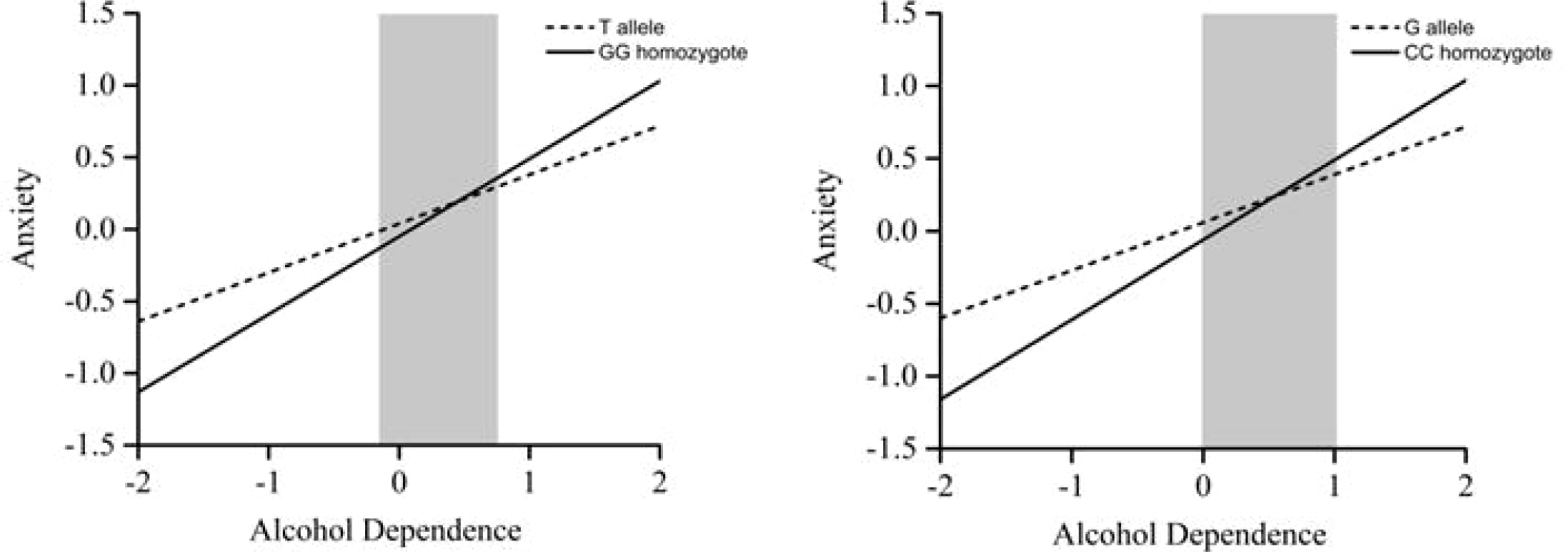
RoS test for alcohol dependence and anxiety by OXT genotype in individuals living with family. Region of significance (RoS) test showing the association between alcohol dependence and anxiety for rs2740210 (left panel) and rs2740209 (right panel) allelic groups in individuals cohabiting with family. Each line represents a genotype group, with the gray shaded area indicating the 95% confidence interval of the crossover point C along the alcohol dependence axis. For individuals living with family, the 95% CI of C ranged from −0.16 to 0.92 for rs2740210 and from −0.01 to 1.02 for rs2740209.

Among individuals living with family, two significant interaction effects emerged: Alcohol dependence × rs2740210 (*p* = 0.02), and alcohol dependence × rs2740209 (*p* = 0.03). Region-of-significance (RoS) analyses showed that individuals carrying the rs2740210 GG homozygote and/or the rs2740209 CC homozygote exhibited significantly higher anxiety levels compared with T-allele carriers (rs2740210) or G-allele carriers (rs2740209), respectively, when standardized alcohol dependence scores exceeded 0.92. No significant interaction was found for rs6133010 (*p* = 0.30). For individuals not living with family, no significant interaction effects were observed (rs2740210: *p* = 0.84; rs2740209: *p* = 0.64; rs6133010: *p* = 0.87). Overall, these findings support a diathesis–stress model, suggesting that these genotypes function as vulnerability factors under conditions of elevated environmental stress (**Figure 2**).

#### Interactions between *OXT* genotyping and alcohol-dependence on anxiety in the different marital status

We conducted hierarchical regression analysis to examine the interaction between *OXT* genotyping and alcohol dependence on anxiety, separately for individuals with different marital status (married vs. unmarried).

There was a significant main effect of alcohol dependence on anxiety in both married and unmarried individuals (married: |*β*|s = 0.42 to 0.55, *p*(s) < 0.001; unmarried: |*β*|s = 0.51 to 0.55, *p*(s) < 0.001), indicating that higher alcohol dependence was associated with higher levels of anxiety. However, there were no significant main effects of *OXT* genotyping (rs2740210, rs2740209, rs6133010) on anxiety across marital status groups (married: |*β*|s = 0.02 to 0.08, *p*(s) > 0.05; unmarried: |*β*|s = 0.02 to 0.14, *p*(s) > 0.05).

**Figure 3:**
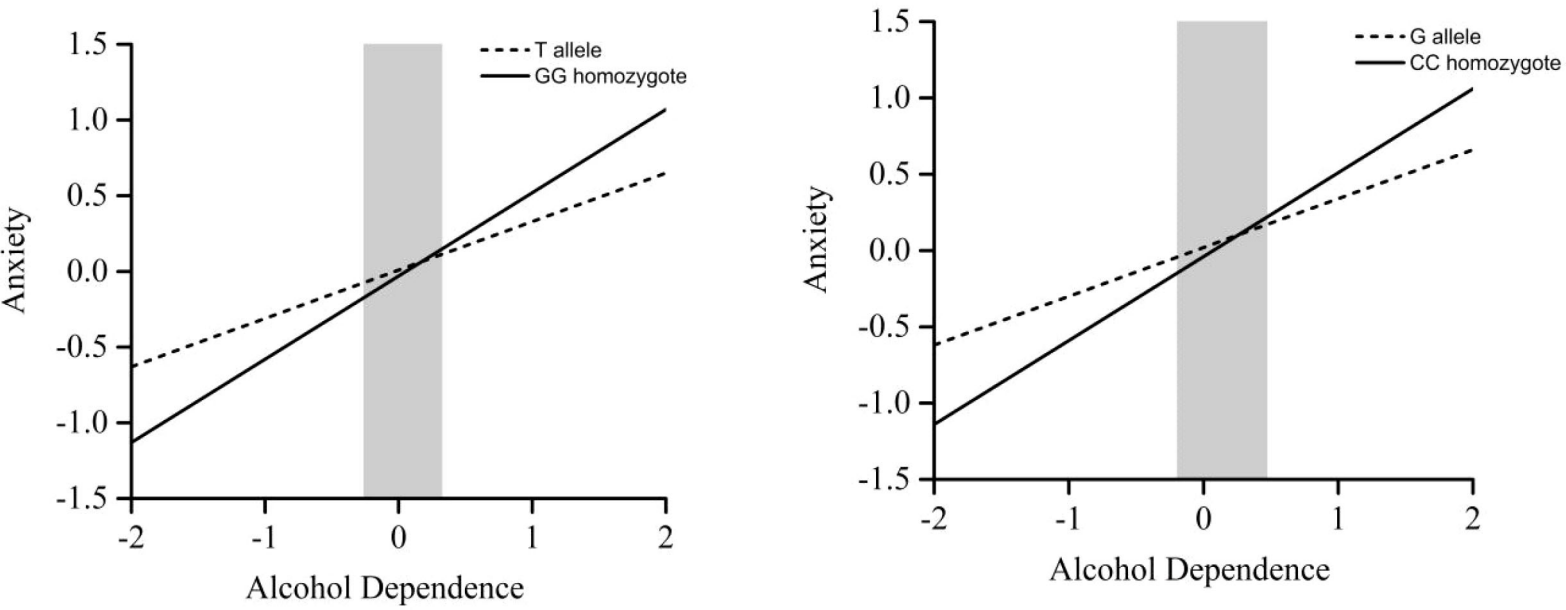
RoS test for alcohol dependence and anxiety by OXT genotype in married individuals. Region of significance (RoS) test showing the association between alcohol dependence and anxiety for rs2740210 (left panel) and rs2740209 (right panel) allelic groups in married individuals. Each line represents a genotype group, with the gray shaded area indicating the 95% confidence interval of the crossover point C along the alcohol dependence axis. For married individuals, the 95% CI of C ranged from −0.27 to 0.60 for rs2740210 and from −0.20 to 0.68 for rs2740209. These revisions ensure that the titles and legends precisely match the content of the figures and maintain consistent SNP naming throughout the manuscript.

Among married individuals, significant interaction effects were observed for both rs2740210 (*p* = 0.03) and rs2740209 (*p* = 0.03). Region-of-significance (RoS) analyses indicated that married individuals carrying the rs2740210 GG homozygote exhibited greater anxiety symptoms relative to T-allele carriers when standardized alcohol dependence scores exceeded 0.60. Likewise, those with the rs2740209 CC homozygote showed higher anxiety compared with G-allele carriers when standardized alcohol dependence scores exceeded 0.68. No significant interaction effect was found for rs6133010 (*p* = 0.46). For unmarried individuals, no significant interaction effects between *OXT* genotyping and alcohol dependence were observed (*p* > 0.05) (see **Figure 3**).

### Interactions between *OXT* genotyping and alcohol-dependence on depression

#### Interactions between *OXT* genotyping and alcohol-dependence on depression in the different living styles

We conducted hierarchical regression analysis to examine the interaction between *OXT* genotyping and alcohol dependence on depression, separately for individuals living with family (yes vs. no).

There was a significant main effect of alcohol dependence on depression across living styles (living with family: |*β*|s = 0.16 to 0.24, *p*(s) < 0.001; living without family: |*β*|s = 0.36, *p*(s) < 0.001). However, no significant main effects of *OXT* genotyping (rs2740210, rs2740209, rs6133010) on depression were found (living with family: |*β*|s = 0.01 to 0.03, *p*(s) > 0.05; living without family: |*β*|s = 0.02 to 0.08, *p*(s) > 0.05).

No significant interaction effects between *OXT* genotyping and alcohol dependence on depression were observed for individuals living with family (rs2740210: *p* = 0.52; rs2740209: *p* = 0.33; rs6133010: *p* = 0.63) or for those living without family (rs2740210: *p* = 0.30; rs2740209: *p* = 0.38; rs6133010: *p* = 0.25).

#### Interactions between *OXT* genotyping and alcohol-dependence on depression in the different marital status

We conducted hierarchical regression analysis to examine the interaction between *OXT* genotyping and alcohol dependence on depression, separately for individuals based on marital status (married vs. unmarried).

There was a significant main effect of alcohol dependence on depression across marital status groups (married: |*β*|s = 0.21 to 0.22, *p*(s) < 0.001; unmarried: |*β*|s = 0.23 to 0.24, *p*(s) < 0.05). However, no significant main effects of *OXT* genotyping (rs2740210, rs2740209, rs6133010) on depression were found (married: |*β*|s = 0.01 to 0.05, *p*(s) > 0.05; unmarried: |*β*|s = 0.04 to 0.06, *p*(s) > 0.05).

No significant interaction effects between *OXT* genotyping and alcohol dependence on depression were observed for married (rs2740210: *p* = 0.67; rs2740209: *p* = 0.55; rs6133010: *p* = 0.23) or unmarried individuals (rs2740210: *p* = 0.56; rs2740209: *p* = 0.71; rs6133010: *p* = 0.60).

### Reparameterized regression analysis

In order to examine the specific pattern of rs2740210 × Alcohol dependence, we conducted reparameterized regression analysis using the models adapted from Widaman et al. (43), as outlined in **Table 4**.

**Table 4:** Results for re-parameterized regression models examining rs2740210 × alcohol dependence interactions on anxiety.

Parameter	Live with family				Married			
	Differential susceptibility		Diathermy-stress		Differential susceptibility		Diathesis-stress	
	Strong: model A	Weak: model B	Strong: model C	Weak: model D	Strong: model A	Weak: model B	Strong: model C	Weak: model D
B_0_	-0.42 (0.40)	-0.25 (0.45)	-0.29 (0.41)	0.23 (0.39)	-0.66 (0.41)	-0.67 (0.45)	-0.53 (0.43)	-0.09 (0.41)
B_1_	0.00 (--)^a^	0.34^***^ (0.07)	0.00 (--)^a^	0.39^***^ (0.06)	0.00 (--)^a^	0.32^***^ (0.07)	0.00 (--)^a^	0.40^***^ (0.06)
C	0.15 (0.19)	0.45 (0.55)	1.56 (--)^a^	1.56 (--)^a^	0.03 (0.20)	0.17 (0.48)	1.56 (--)^a^	1.56 (--)^a^
95%CI of C	[-0.22, 052]	[-0.63, 1.53]	(--)	(--)	[-0.36, 042]	[-0.77, 1.11]	(--)	(--)
B_2_	0.54^***^ (0.07)	0.54^***^ (0.07)	0.27^***^ (0.05)	0.49^***^ (0.06)	0.54^***^ (0.08)	0.54^***^ (0.07)	0.25^***^ (0.05)	0.48^***^ (0.06)
B_3_	0.01 (0.01)	0.01 (0.01)	0.01 (0.01)	0.01 (0.01)	0.01 (0.01)	0.01 (0.01)	0.01 (0.01)	0.01 (0.01)
B_4_	0.03 (0.02)	0.02 (0.02)	0.03 (0.02)	0.02 (0.02)	0.04 (0.02)	0.03 (0.02)	0.04 (0.02)	0.04 (0.02)
R²	0.14	0.21	0.09	0.21	0.16	0.21	0.08	0.21
F (df)	14.51^***^ (4, 324)	21.51^***^ (5, 323)	10.41^***^ (3, 325)	21.05^***^ (4, 324)	14.13^***^ (4, 294)	19.91^***^ (5, 293)	8.63^***^ (3, 295)	19.10^***^ (4, 294)
F vs.c (df)	24.37^***^ (1, 324)	24.37^***^ (2, 323)	(--)	48.26^***^ (1, 324)	28.07^***^ (1, 294)	24.57^***^ (2, 293)	(--)	46.35^***^ (1, 294)
F vs.d (df)	(--)	1.43 (1, 323)	48.26^***^ (1, 323)	(--)	(--)	2.50 (1, 293)	46.35^***^ (1, 293)	(--)
AIC	890.45	869.19	912.48	868.65	806.94	789.84	832.36	790.45
BIC	913.23	895.76	931.46	891.43	829.14	815.74	850.84	812.65

Model:Anxiety= (*OXT*.rs2740210=GT/TT) (B_0_+B_1_ (X_MAST_-C))+ (*OXT*.rs2740210=GG) (B_0_+B_2_ (X_MAST_-C))+B_3_X_marital status_+B_4_X_livingstyle_+E;.

^a^ Parameter fixed at reported value; SE is not applicable, so is listed as –. *F* versus a stands for *F* tests of the difference in R^2^ for a given model versus the strong differential susceptibility model;*p<0.05, **p<0.01, ***p<0.001.

AIC, Akaike Information Criterion; BIC, Bayesian Information Criterion.

For the subjects living with family, the weak diathesis-stress model (model D) with C = 1.56 accounted for a significant amount of variance in anxiety (*R*^2^ = 0.21, *p* < 0.001). In this model, the slopes for alcohol dependence were significant in the T allele group (*B*_1_ = 0.39, SE = 0.06, *p* < 0.001) and the GG homozygote group (*B*_2_ = 0.49, *SE* = 0.06, *p* < 0.001). Furthermore, the weak diathesis-stress model explained more variance (*ΔR*^2^ = 0.12, *p* < 0.001) by adding one more parameter compared to the strong diathesis-stress model, while it explained the same variance by reducing one more parameter compared to the weak differential susceptibility model (*ΔR*^2^ < 0.001, *p* > 0.05). Additionally, the weak diathesis-stress model exhibited smaller AIC and BIC values when compared with the strong differential susceptibility model. In summary, all the statistical indexes support the weak diathesis-stress model, indicating that the T allele is a non-risk allele and the GG homozygote is a risk homozygote.

For the married subjects, the weak diathesis-stress model with C = 1.56 provided a strong fit to the data (*R*^2^ = 0.21, *p* < 0.001). In this model, the slopes for alcohol dependence were significant in the T allele group (*B*_1_ = 0.40, *SE* = 0.06, *p* < 0.001) and the GG homozygote group (*B*_2_ = 0.48, *SE* = 0.06, *p* < 0.001). Furthermore, the weak diathesis-stress model demonstrated a better fit to the data compared to other models, indicating that the GG homozygote is a risk homozygote and the T allele is a non-risk allele in relation to anxiety.

Regarding rs2740209 × Alcohol dependence, we also conducted reparameterized regression analysis using the regression models, as outlined in **Table 5**.

**Table 5:** Results for re-parameterized regression models examining rs2740209 × alcohol dependence interactions on anxiety.

Parameter	Live with family				Married			
	Differential susceptibility		Diathesis-stress		Differential susceptibility		Diathesis-stress	
	Strong: model A	Weak: model B	Strong: model C	Weak: model D	Strong: model A	Weak: model B	Strong: model C	Weak: model D
B_0_	-0.35 (0.40)	-0.18 (0.45)	-0.19 (0.41)	0.26 (0.39)	-0.58 (0.42)	-0.59 (0.45)	-0.43 (0.43)	-0.06 (0.40)
B_1_	0.00 (--)^a^	0.33^***^ (0.07)	0.00 (--)^a^	0.38^***^ (0.06)	0.00 (--)^a^	0.32^***^ (0.07)	0.00 (--)^a^	0.39^***^ (0.06)
C	0.20 (0.19)	0.54 (0.53)	1.56 (--)^a^	1.56 (--)^a^	0.08 (0.20)	0.27 (0.48)	1.56 (--)^a^	1.56 (--)^a^
95%CI of C	[-0.17, 0.57]	[-0.50, 1.58]	(--)	(--)	[-0.31, 0.47]	[-0.67, 1.21]	(--)	(--)
B_2_	0.54^***^ (0.07)	0.54^***^ (0.07)	0.28^***^ (0.05)	0.50^***^ (0.06)	0.54^***^ (0.08)	0.54^***^ (0.07)	0.26^***^ (0.05)	0.48^***^ (0.06)
B_3_	0.01 (0.01)	0.01 (0.01)	0.01 (0.01)	0.01 (0.01)	0.01 (0.01)	0.01 (0.01)	0.01 (0.01)	0.01 (0.01)
B_4_	0.02 (0.02)	0.02 (0.02)	0.03 (0.02)	0.02 (0.02)	0.03 (0.02)	0.03 (0.02)	0.04 (0.02)	0.03 (0.02)
R²	0.16	0.21	0.10	0.21	0.16	0.21	0.09	0.21
F (df)	15.52^***^ (4, 324)	21.90^***^ (5, 323)	11.91^***^ (3.325)	21.45^***^ (4, 324)	14.39^***^ (4,.294)	19.99^***^ (5, 293)	9.47^***^ (3, 295)	19.29^***^ (4, 294)
F vs.c (df)	23.64^***^ (1, 324)	23.25^***^ (2, 323)	(--)	45.04^***^ (1, 324)	26.50^***^ (1, 294)	23.38^***^ (2, 293)	(--)	44.39^***^ (1, 294)
F vs.d (df)	(--)	1.39 (1, 323)	45.04^***^ (1, 323)	(--)	(--)	2.19 (1, 293)	44.39^***^ (1, 293)	(--)
AIC	886.99	867.92	908.34	867.35	806.07	789.60	830.04	789.84
BIC	909.77	894.50	927.32	890.13	828.27	815.50	848.84	812.05

^a^ Parameter fixed at reported value; SE is not applicable, so is listed as –. *F* versus a stands for *F* tests of the difference in R^2^ for a given model versus the strong differential susceptibility model;*p<0.05, **p<0.01, ***p<0.001.

AIC, Akaike Information Criterion; BIC, Bayesian Information Criterion.

For the subjects living with family, the weak diathesis-stress model (model D) with C = 1.56 accounted for a significant amount of variance in anxiety (*R*^2^ = 0.21, *p* < 0.001). In this model, the slopes for alcohol dependence were significant in the G allele group (*B*_1_ = 0.38, *SE* = 0.06, *p* < 0.001) and the CC homozygote group (*B*_2_ = 0.50, *SE* = 0.06, *p* < 0.001). Furthermore, the weak diathesis-stress model explained more variance (*ΔR*^2^ = 0.11, *p* < 0.001) by adding one more parameter compared to the strong diathesis-stress model, while it explained the same variance by reducing one more parameter compared to the weak differential susceptibility model (*ΔR*^2^ < 0.001, *p* > 0.05). Additionally, the weak diathesis-stress model exhibited smaller AIC and BIC values when compared with the strong differential susceptibility model. In summary, all the statistical indexes support the weak diathesis-stress model, indicating that the G allele is a non-risk allele and the CC homozygote is a risk homozygote.

For the married subjects, the weak diathesis-stress model with C = 1.56 provided a strong fit to the data (*R*^2^ = 0.21, *p* < 0.001). In this model, the slopes for alcohol dependence were significant in the G allele group (*B*_1_ = 0.39, *SE* = 0.06, *p* < 0.001) and the CC homozygote group (*B*_2_ = 0.48, *SE* = 0.06, *p* < 0.001). Furthermore, the weak diathesis-stress model demonstrated a better fit to the data compared to other models, indicating that the CC homozygote is a risk homozygote and the G allele is a non-risk allele in relation to anxiety.

## Discussion

The present study yields several key findings regarding the interplay between genetic predisposition and environmental context and its association to negative emotions during acute alcohol withdrawal. Furthermore, the nature of this G × E interaction was evaluated in relation to two theoretical frameworks: the diathesis-stress model and the differential susceptibility model. The findings provide several key insights.

Firstly, as expected, we found significant concurrent associations between alcohol dependence and depression or anxiety symptoms during acute alcohol withdrawal. Further analysis revealed that the severity of alcohol dependence was associated with an increased risk of depression and anxiety symptoms, providing support for the first hypothesis and aligning with previous studies (44). In these results underscore the substantial psychological burden experienced during acute withdrawal and highlight dependence severity as a key clinical predictor of emotional distress in this high-stress transitional period. When examining the associations between *OXT* polymorphisms (rs2740210, rs6133010, rs2740209) and emotional symptoms, we unexpectedly observed a significant association between rs6133010 and anxiety severity. This finding has not been previously reported. Considering the location of these three variants in the genomic structure of the *OXT* gene, rs6133010 may be directly involved in the regulation of transcription factor binding. However, further molecular biological experiments are needed to confirm this.

Secondly, in the anxiety model, *OXT* polymorphism (rs2740210 and rs2740209) significantly mediated the association between alcohol dependence and anxiety symptoms during acute alcohol withdrawal, confirming hypothesis 2a and supporting the diathesis-stress theory. Furthermore, all the indexes in the re-parameterized regressions indicated that *OXT* polymorphism (rs2740210 and rs2740209) × alcohol dependence interactions were consistent with the weak diathesis-stress model among male adults with anxiety symptoms. Specifically, compared to adults with the G allele of *OXT*.rs2740209 or the T allele of *OXT*.rs2740210, those with GG homozygote of rs2740210 and CC homozygote of rs2740209 reported more anxiety symptoms when experiencing a more severe level of alcohol dependence. Previous studies suggest that the interaction between *OXTR* polymorphism and early life stress can predict the level of anxiety or depression (24). Additionally, one report demonstrated that carriers of the OXT rs2740210 CA/AA (corresponding to GT/TT in the present study) polymorphism exposed to maternal verbally aggressive behavior are associated with general anxiety at ages 5-6 (45). The downstream variant rs2740210 is located in genomic regions that may potentially influence gene regulation. Although direct functional evidence is lacking in the current study, such regions are commonly involved in epigenetic mechanisms, including DNA methylation—a key pathway in gene–environment (G × E) interactions (46). Therefore, our study provides new evidence for the mediating function of *OXT* polymorphism in the association between current stress and anxiety symptoms.

Thirdly, in the depression model, there was no interaction between *OXT* polymorphism and alcohol dependence, which contradicts hypothesis 2b. Several reasons may help explain or interpret this unexpected finding. Although increased expression levels of OXT were found in post-mortem hypothalamic tissue from depressed patients, it remains unclear whether such alterations represent causes or consequences (47). Additionally, anxiety and depression may differ biologically, including in terms of functional networks and susceptibility genes (48). Therefore, alcohol dependence may interact with other specific genes in predicting the occurrence of depression, rather than with OXT.

Fourthly, consistent with the third hypothesis, the mediating effects of *OXT* polymorphism on the association between alcohol dependence level and anxiety were only observed among adults living with family or married adults. For adults living without family or unmarried adults, no significant interaction effect of *OXT* genotyping and alcohol dependence was found. This suggests that the effects of OXT on anxiety in humans occur in a context-dependent manner. Previous studies have shown that OXT buffers anxiety in an affiliative context but not in a non-affiliative context (49). These findings provide additional evidence for the social salience hypothesis of OXT, suggesting that the multiple physiological roles of OXT in social bonds and anxiety can be integrated into the overall stress management process.

The current study makes valuable contributions to the existing literature by providing important insights into the underlying mechanisms of negative emotions (depression and anxiety) in adults during alcohol withdrawal. It possesses several notable strengths.

Firstly, to the best of our knowledge, this study is the first to examine the interactions between genetic and environmental factors (G × E effects) on both depression and anxiety symptoms during alcohol withdrawal in adults. By doing so, it provides preliminary evidence for distinct G × E interactions specific to these two types of negative emotions during alcohol withdrawal.

Secondly, by focusing on the level of alcohol dependence, this study comprehensively tests whether the G × E interactions align with either the diathesis-stress model or the differential susceptibility model. This approach allows for a more thorough understanding of the interaction patterns and their implications.

Thirdly, this research provides support for the context-dependent effect of oxytocin and reinforces the significance of considering contextual factors when examining the role of OXT as a biological substrate underlying social bonding and stress regulatory processes. It also highlights the importance of considering contextual factors when studying the effects of OXT administration, particularly in patients with psychiatric disorders.

A critical nuance of our findings is that not all carriers of the putative “risk” genotypes (GG or CC) developed elevated anxiety; rather, the genetic effect was contingent upon environmental context. This observation aligns with the weak diathesis-stress framework, which posits that all individuals are sensitive to environmental influences, albeit to varying degrees. From a practical standpoint, this implies that genetic risk is not deterministic but probabilistic, becoming clinically relevant primarily under specific relational conditions. From a clinical perspective, male adults with alcohol use disorder (AUD) who are married or living with family should be routinely monitored for anxiety during acute withdrawal. Where resources permit, genetic testing could further identify individuals carrying risk alleles who may require targeted interventions.

Several limitations in this study should be highlighted, suggesting potential avenues for future investigations. First, the cross-sectional design precludes causal inference regarding the temporal dynamics among alcohol dependence, genetic susceptibility, and emotional symptoms. Second, the sample comprised exclusively Han Chinese males, restricting the generalizability of findings to females and other ethnic populations. While this homogeneity strengthens internal validity, future studies should explore whether similar mechanisms operate in women and more diverse cultural groups. Third, social context was measured using dichotomous indicators (marital status and cohabitation), which may not fully capture the quality or perceived level of social support. For example, the “unmarried” group may have included individuals in stable cohabiting relationships, thus further research is warranted. Additionally, in the present study, alcohol dependence and negative emotions were assessed using single informants, which may introduce bias and limited perspectives. Future research should aim to incorporate multiple informants to provide a more comprehensive understanding of these constructs. The genotypes of rs2740210 and rs2740209 were highly concordant, with 20 of 414 individuals (4.8%) showing discordant genotypes, reflecting strong linkage between these SNPs. Consequently, the results of these two SNPs are largely redundant, and their joint reporting provides a more concise presentation of the findings. Furthermore, withdrawal severity was not assessed using standardized instruments (e.g., CIWA-Ar), nor was alcohol craving measured—both of which may serve as important covariates in future models. Finally, future research should expand the scope of genetic investigation to include a more comprehensive panel of *OXT* and *OXTR* variants, thereby enabling a systematic characterization of how *OXT*-related genetic variation contributes to the pathophysiology of psychiatric disorders.

In conclusion, this study demonstrates that variation in the *OXT* gene—particularly at rs2740210 and rs2740209—interacts with alcohol dependence to shape anxiety symptoms during acute withdrawal in a context-dependent manner. These findings have translational implications: in clinical settings, male patients with alcohol dependence could be preliminarily stratified based on their *OXT* genotype and social context to identify those at heightened risk for withdrawal-related anxiety. Such an approach could facilitate earlier psychological or oxytocin-targeted interventions, potentially mitigating the escalation of anxiety, reducing relapse rates, and alleviating the overall burden on healthcare systems. Collectively, this study underscores the value of integrating genetic and environmental profiling to advance personalized treatment strategies for alcohol use disorder. Our findings demonstrate that G × E interactions modulate individual differences in emotional reactivity to stress, thereby informing more nuanced etiological models of psychiatric disorders. An integrative framework that jointly accounts for genetic susceptibility and environmental exposure holds significant promise for refining risk stratification, developing targeted interventions, which may ultimately enhancing prevention efforts and clinical outcomes across stress-related psychiatric conditions.

## Data Availability

The raw data supporting the conclusions of this article will be made available by the authors, without undue reservation.
