# Supplementary figures and images for: Context-dependent interaction between oxytocin gene polymorphisms and alcohol dependence in modulating negative emotions during acute alcohol withdrawal in adult males
Source: Front Psychiatry. 2026 May 15;17:1680226. doi: 10.3389/fpsyt.2026.1680226 (PMC13219007; doi:10.3389/fpsyt.2026.1680226)

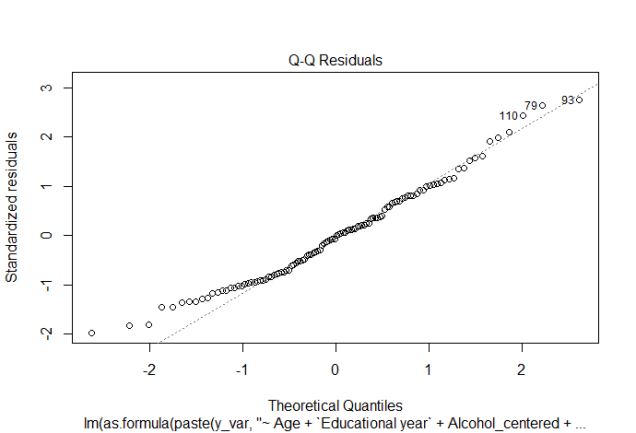


**
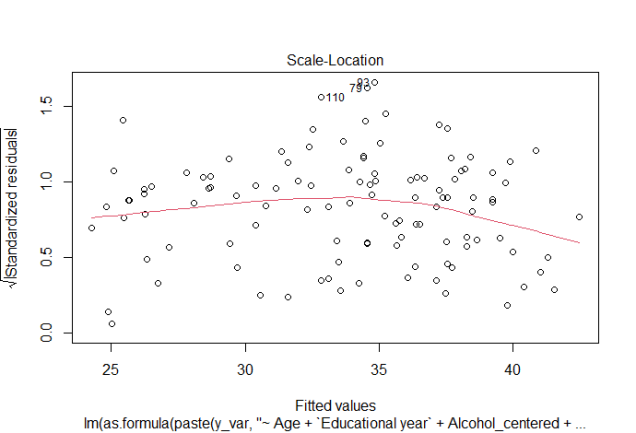
**

Supplement: Supplementary file 1 [file SupplementaryFile1.docx]
