# Supplementary material for: Context-dependent interaction between oxytocin gene polymorphisms and alcohol dependence in modulating negative emotions during acute alcohol withdrawal in adult males
Source: Front Psychiatry. 2026 May 15;17:1680226. doi: 10.3389/fpsyt.2026.1680226 (PMC13219007; doi:10.3389/fpsyt.2026.1680226)
Supplement: Supplementary file 2 [file Table1.docx]

### Table1 Effects of rs2740210, Alcohol Dependence, and Their Interaction on Anxiety by Living Situation

| **Variables** | **living with family** | | | | | **living without family** | | | | |
| --- | --- | --- | --- | --- | --- | --- | --- | --- | --- | --- |
|  | ***ΔR^2^*** | ***B*(*SE*)** | ***β*** | ***t*** | ***p*** | ***ΔR^2^*** | ***B*(*SE*)** | ***β*** | ***t*** | ***p*** |
| Age | 0.01 | 0.01(0.01) | 0.02 | 0.29 | 0.770 | 0.03 | 0.01(0.01) | 0.05 | 0.43 | 0.670 |
| Educational Years |  | 0.04(0.02) | 0.11 | 1.78 | 0.070 |  | 0.07(0.04) | 0.20 | 1.62 | 0.110 |
| Alcohol Dependence | 0.19 | 0.54(0.07) | 0.54 | 8.74 | **<0.001** | 0.21 | 0.48(0.10) | 0.48 | 4.64 | **<0.001** |
| rs2740210 |  | 0.09(0.10) | 0.04 | 0.89 | 0.370 |  | -0.15(0.20) | -0.08 | -0.75 | 0.450 |
| Alcohol Dependenc×rs2740210 | 0.01 | -0.20(0.10) | -0.14 | -2.00 | **0.020** | <0.001 | -0.04(0.20) | -0.03 | -0.21 | 0.840 |

### Table2 Effects of rs2740209, Alcohol Dependence, and Their Interaction on Anxiety by Living Situation

| **Variables** | **living with family** | | | | | **living without family** | | | | |
| --- | --- | --- | --- | --- | --- | --- | --- | --- | --- | --- |
|  | ***ΔR^2^*** | ***B*(*SE*)** | ***β*** | ***t*** | ***p*** | ***ΔR^2^*** | ***B*(*SE*)** | ***β*** | ***t*** | ***p*** |
| Age | 0.01 | 0.01(0.01) | 0.02 | 0.29 | 0.770 | 0.03 | 0.01(0.01) | 0.05 | 0.43 | 0.670 |
| Educational Years |  | 0.04(0.02) | 0.11 | 1.78 | 0.070 |  | 0.07(0.04) | 0.20 | 1.62 | 0.110 |
| Alcohol Dependence | 0.19 | 0.44(0.05) | 0.44 | 8.75 | **<0.001** | 0.21 | 0.49(0.10) | 0.49 | 4.67 | **<0.001** |
| rs2740209 |  | 0.12(0.10) | 0.06 | 1.18 | 0.240 |  | -0.16(0.20) | -0.08 | -0.80 | -0.800 |
| Alcohol Dependenc×rs2740209 | 0.01 | -0.21(0.10) | -0.15 | -2.16 | **0.030** | <0.001 | 0.10(.20) | 0.06 | 0.47 | 0.640 |

### Table3 Effects of rs6133010, Alcohol Dependence, and Their Interaction on Anxiety by Living Situation

| **Variables** | **living with family** | | | | | **living without family** | | | | |
| --- | --- | --- | --- | --- | --- | --- | --- | --- | --- | --- |
|  | ***ΔR^2^*** | ***B*(*SE*)** | ***β*** | ***t*** | ***p*** | ***ΔR^2^*** | ***B*(*SE*)** | ***β*** | ***t*** | ***p*** |
| Age | 0.01 | 0.01(0.01) | 0.02 | 0.29 | 0.770 | 0.03 | 0.01(0.01) | 0.05 | 0.43 | 0.67 |
| Educational Years |  | 0.04(0.02) | 0.11 | 1.78 | 0.070 |  | 0.07(0.04) | 0.20 | 1.62 | 0.11 |
| Alcohol Dependence | 0.19 | 0.44(0.05) | 0.44 | 8.66 | **<0.001** | 0.25 | 0.43(0.10) | 0.43 | 4.13 | **<0.001** |
| rs6133010 |  | -0.13(0.10) | -0.07 | -1.31 | 0.190 |  | -0.44(0.20) | -0.22 | -2.27 | **0.03** |
| Alcohol Dependence×Rs6133010 | 0.002 | 0.10(0.10) | 0.08 | 1.04 | 0.300 | <0.001 | 0.03(0.20) | 0.02 | 0.17 | 0.87 |

### Table 4 Effects of rs2740210, Alcohol Dependence, and Their Interaction on Depression by Living Situation

| **Variables** | **living with family** | | | | | **living without family** | | | | |
| --- | --- | --- | --- | --- | --- | --- | --- | --- | --- | --- |
|  | ***ΔR^2^*** | ***B*(*SE*)** | ***β*** | ***t*** | ***p*** | ***ΔR^2^*** | ***B*(*SE*)** | ***β*** | ***t*** | ***p*** |
| Age | 0.001 | -0.003(0.01) | -0.03 | -0.49 | 0.620 | 0.01 | 0.01(0.01) | 0.11 | 0.92 | 0.360 |
| Educational Years |  | 0.001(0.02) | 0.002 | 0.04 | 0.970 |  | 0.02(0.04) | 0.04 | 0.35 | 0.730 |
| Alcohol Dependence | 0.04 | 0.19(0.05) | 0.19 | 3.43 | **<0.001** | 0.12 | 0.36(0.11) | 0.36 | 3.27 | **0.002** |
| rs2740210 |  | -0.06(0.11) | -0.03 | -0.51 | 0.610 |  | -0.16（0.21） | -0.08 | -0.74 | 0.460 |
| Alcohol Dependenc×rs2740210 | 0.002 | -0.07(0.11) | -0.05 | -0.64 | 0.520 | <0.001 | 0.22(0.21) | 0.15 | 1.04 | 0.300 |

### Table5 Effects of rs2740209, Alcohol Dependence, and Their Interaction on Depression by Living Situation

| **Variables** | **living with family** | | | | | **living without family** | | | | |
| --- | --- | --- | --- | --- | --- | --- | --- | --- | --- | --- |
|  | ***ΔR^2^*** | ***B*(*SE*)** | ***β*** | ***t*** | ***p*** | ***ΔR^2^*** | ***B*(*SE*)** | ***β*** | ***t*** | ***p*** |
| Age | 0.01 | -0.003(0.01) | -0.03 | -0.49 | 0.620 | 0.01 | 0.01(0.01) | 0.11 | 0.92 | 0.360 |
| Educational Years |  | 0.001(0.02) | 0.002 | 0.04 | 0.970 |  | 0.02(0.04) | 0.04 | 0.35 | 0.730 |
| Alcohol Dependence | 0.04 | 0.19(0.05) | 0.19 | 3.44 | **<0.001** | 0.12 | 0.36(0.11) | 0.36 | 3.24 | **0.002** |
| rs2740209 |  | -0.03(0.11) | -0.02 | -0.31 | 0.750 |  | -0.05(0.22) | -0.03 | -0.24 | 0.810 |
| Alcohol Dependenc×rs2740209 | 0.002 | -0.11(0.11) | -0.07 | -0.97 | 0.330 | <0.001 | 0.19(0.22) | 0.12 | 0.87 | 0.380 |

### Table 6 Effects of rs6133010, Alcohol Dependence, and Their Interaction on Depression by Living Situation

| **Variables** | **living with family** | | | | | **living without family** | | | | |
| --- | --- | --- | --- | --- | --- | --- | --- | --- | --- | --- |
|  | ***ΔR^2^*** | ***B*(*SE*)** | ***β*** | ***t*** | ***p*** | ***ΔR^2^*** | ***B*(*SE*)** | ***β*** | ***t*** | ***p*** |
| Age | 0.001 | -0.003(0.01) | -0.03 | -0.49 | 0.620 | 0.01 | 0.01(0.01) | 0.11 | 0.92 | 0.360 |
| Educational Years |  | 0.001(0.02) | 0.002 | 0.04 | 0.970 |  | 0.02(0.04) | 0.04 | 0.35 | 0.730 |
| Alcohol Dependence | 0.04 | 0.16(0.08) | 0.16 | 3.43 | **<0.001** | 0.11 | 0.36(0.11) | 0.36 | 3.18 | **0.002** |
| rs6133010 |  | -0.01(0.11) | -0.01 | -0.11 | 0.920 |  | 0.03(0.21) | 0.02 | 0.15 | 0.880 |
| Alcohol Dependenc×rs6133010 | 0.002 | 0.05(0.11) | 0.04 | 0.48 | 0.630 | <0.001 | 0.26(0.22) | 0.16 | 1.16 | 0.250 |

### Table 7 Effects of rs2740210, Alcohol Dependence, and Their Interaction on Anxiety by Marital Status

| **Variables** | **married** | | | | | **unmarried** | | | | |
| --- | --- | --- | --- | --- | --- | --- | --- | --- | --- | --- |
|  | ***ΔR^2^*** | ***B*(*SE*)** | ***β*** | ***t*** | ***p*** | ***ΔR^2^*** | ***B*(*SE*)** | ***β*** | ***t*** | ***p*** |
| Age | 0.02 | 0.01(0.01) | 0.04 | 0.72 | 0.470 | 0.01 | -0.01(0.01) | -0.07 | -0.58 | 0.570 |
| Educational Years |  | 0.05(0.02) | 0.13 | 2.26 | **0.020** |  | 0.02(0.04) | 0.06 | 0.49 | 0.630 |
| Alcohol Dependence | 0.18 | 0.55(0.07) | 0.55 | 8.23 | **<0.001** | 0.25 | 0.54(0.09) | 0.54 | 5.93 | **<0.001** |
| rs2740210 |  | 0.04(0.10) | 0.02 | 0.35 | 0.730 |  | 0.04(0.17) | 0.02 | 0.23 | 0.820 |
| Alcohol Dependenc×rs2740210 | 0.01 | -0.22(0.10) | -0.16 | -2.13 | **0.030** | <0.001 | -0.06(0.17) | -0.04 | -0.38 | 0.710 |

### Table 8 Effects of rs2740209, Alcohol Dependence, and Their Interaction on Anxiety by Marital Status

| **Variables** | **married** | | | | | **unmarried** | | | | |
| --- | --- | --- | --- | --- | --- | --- | --- | --- | --- | --- |
|  | ***ΔR^2^*** | ***B*(*SE*)** | ***β*** | ***t*** | ***p*** | ***ΔR^2^*** | ***B*(*SE*)** | ***β*** | ***t*** | ***p*** |
| Age | 0.02 | 0.01(0.01) | 0.04 | 0.72 | 0.470 | 0.01 | -0.01(0.01) | -0.07 | -0.58 | 0.570 |
| Educational Years |  | 0.05(0.02) | 0.13 | 2.26 | **0.020** |  | 0.02(0.04) | 0.06 | 0.49 | 0.630 |
| Alcohol Dependence | 0.18 | 0.43(0.05) | 0.43 | 8.25 | **<0.001** | 0.25 | 0.54(0.09) | 0.54 | 5.89 | **<0.001** |
| rs2740209 |  | 0.06(0.10) | 0.03 | 0.57 | 0.570 |  | 0.04(0.17) | 0.02 | 0.24 | 0.820 |
| Alcohol Dependenc×rs2740209 | 0.01 | -0.22(0.10) | -0.16 | -2.13 | **0.030** | <0.001 | -0.02(0.17) | -0.02 | -0.13 | 0.890 |

### Table 9 Effects of rs6133010, Alcohol Dependence, and Their Interaction on Anxiety by Marital Status

| **Variables** | **married** | | | | | **unmarried** | | | | |
| --- | --- | --- | --- | --- | --- | --- | --- | --- | --- | --- |
|  | ***ΔR^2^*** | ***B*(*SE*)** | ***β*** | ***t*** | ***p*** | ***ΔR^2^*** | ***B*(*SE*)** | ***β*** | ***t*** | ***p*** |
| Age | 0.02 | 0.01(0.01) | 0.04 | 0.72 | 0.470 | 0.01 | -0.01(0.01) | -0.07 | -0.58 | 0.570 |
| Educational Years |  | 0.05(0.02) | 0.13 | 2.26 | **0.020** |  | 0.02(0.04) | 0.06 | 0.49 | 0.630 |
| Alcohol Dependence | 0.18 | 0.42(0.08) | 0.42 | 8.03 | **<0.001** | 0.26 | 0.51(0.12) | 0.51 | 6.01 | **<0.001** |
| rs6133010 |  | -0.15(0.11) | -0.08 | -1.46 | 0.150 |  | -0.28(0.16) | -0.14 | -1.74 | 0.080 |
| Alcohol Dependenc×rs6133010 | 0.001 | 0.08(0.11) | 0.06 | 0.75 | 0.460 | <0.001 | 0.07(0.17) | 0.05 | 0.44 | 0.660 |

### Table 10 Effects of rs2740210, Alcohol Dependence, and Their Interaction on Depression by Marital Status

| **Variables** | **married** | | | | | **unmarried** | | | | |
| --- | --- | --- | --- | --- | --- | --- | --- | --- | --- | --- |
|  | ***ΔR^2^*** | ***B*(*SE*)** | ***β*** | ***t*** | ***p*** | ***ΔR^2^*** | ***B*(*SE*)** | ***β*** | ***t*** | ***p*** |
| Age | <0.001 | 0.001(0.01) | 0.001 | 0.01 | 0.990 | 0.002 | -0.004(0.01) | -0.04 | -0.34 | 0.730 |
| Educational Years |  | -0.001(0.02) | -0.001 | -0.02 | 0.980 |  | 0.004(0.04) | 0.13 | 0.11 | 0.910 |
| Alcohol Dependence | 0.05 | 0.21(0.06) | 0.21 | 3.73 | **<0.001** | 0.05 | 0.24(0.10) | 0.24 | 2.34 | **0.020** |
| rs2740210 |  | -0.10(0.11) | -0.05 | -0.89 | 0.370 |  | -0.07(0.19) | -0.04 | -0.37 | 0.710 |
| Alcohol Dependence×rs2740210 | <0.001 | -0.05(0.11) | -0.03 | -0.42 | 0.670 | <0.001 | 0.11(0.19) | 0.08 | 0.58 | 0.560 |

### Table 11 Effects of rs2740209, Alcohol Dependence, and Their Interaction on Depression by Marital Status

| **Variables** | **married** | | | | | **unmarried** | | | | |
| --- | --- | --- | --- | --- | --- | --- | --- | --- | --- | --- |
|  | ***ΔR^2^*** | ***B*(*SE*)** | ***β*** | ***t*** | ***p*** | ***ΔR^2^*** | ***B*(*SE*)** | ***β*** | ***t*** | ***p*** |
| Age | <0.001 | 0.001(0.01) | 0.001 | 0.01 | 0.990 | 0.002 | -0.004(0.01) | -0.04 | -0.34 | 0.730 |
| Educational Years |  | -0.001(0.02) | -0.001 | -0.02 | 0.980 |  | 0.004(0.04) | 0.13 | 0.11 | 0.910 |
| Alcohol Dependence | 0.05 | 0.22(0.06) | 0.22 | 3.74 | **<0.001** | 0.05 | 0.23(0.10) | 0.23 | 2.23 | **0.030** |
| rs2740209 |  | -0.09(0.11) | -0.05 | -0.82 | 0.410 |  | 0.08(0.19) | 0.04 | 0.43 | 0.670 |
| Alcohol Dependence×rs2740209 | <0.001 | -0.07(0.12) | -0.05 | -0.60 | 0.550 | <0.001 | 0.07(0.19) | 0.05 | 0.38 | 0.710 |

### Table 12 Effects of rs6133010, Alcohol Dependence, and Their Interaction on Depression by Marital Status

| **Variables** | **married** | | | | | **unmarried** | | | | |
| --- | --- | --- | --- | --- | --- | --- | --- | --- | --- | --- |
|  | ***ΔR^2^*** | ***B*(*SE*)** | ***β*** | ***t*** | ***p*** | ***ΔR^2^*** | ***B*(*SE*)** | ***β*** | ***t*** | ***p*** |
| Age | <0.001 | 0.01(0.01) | 0.01 | 0.23 | 0.820 | 0.002 | -0.004(0.01) | -0.04 | -0.34 | 0.730 |
| Educational Years |  | -0.01(0.02) | -0.02 | -0.35 | 0.730 |  | 0.004(0.04) | 0.13 | 0.11 | 0.910 |
| Alcohol Dependence | 0.05 | 0.22(0.06) | 0.22 | 3.79 | **<0.001** | 0.05 | 0.24(0.10) | 0.24 | 2.29 | **0.020** |
| rs6133010 |  | 0.02(0.12) | 0.01 | 0.15 | 0.870 |  | -0.12(0.19) | -0.06 | -0.66 | 0.510 |
| Alcohol Dependence×rs6133010 | <0.001 | 0.14(0.12) | 0.10 | 1.22 | 0.230 | <0.001 | -0.10(0.19) | -0.07 | -0.52 | 0.600 |
